# Ecotoxicity Assessment of Graphene Oxide by *Daphnia magna* through a Multimarker Approach from the Molecular to the Physiological Level including Behavioral Changes

**DOI:** 10.3390/nano10102048

**Published:** 2020-10-16

**Authors:** Ildikó Fekete-Kertész, Krisztina László, Csilla Terebesi, Benjámin Sándor Gyarmati, Shereen Farah, Rita Márton, Mónika Molnár

**Affiliations:** 1 Environmental Microbiology and Biotechnology Group, Department of Applied Biotechnology and Food Science, Faculty of Chemical Technology and Biotechnology, Budapest University of Technology and Economics, Műegyetem rkp. 3., 1111 Budapest, Hungary; fekete.kertesz.ildiko@mail.bme.hu (I.F.-K.); terebesics@gmail.com (C.T.); ritamarton34@gmail.com (R.M.); 2 Surface Chemistry Group, Department of Physical Chemistry and Materials Science, Faculty of Chemical Technology and Biotechnology, Budapest University of Technology and Economics, Műegyetem rkp. 3., 1111 Budapest, Hungary; klaszlo@mail.bme.hu (K.L.); shereen.farah@mail.bme.hu (S.F.); 3 Soft Matters Group, Department of Physical Chemistry and Materials Science, Faculty of Chemical Technology and Biotechnology, Budapest University of Technology and Economics, Műegyetem rkp. 3., 1111 Budapest, Hungary; bgyarmati@mail.bme.hu

**Keywords:** *Daphnia magna*, feeding activity, graphene oxide, heartbeat rate, nano-ecotoxicology, oxidative stress, recovery studies

## Abstract

The extensive use of engineered nanomaterials, such as graphene oxide (GO), is stimulating research about its potential environmental impacts on the aquatic ecosystem. This study is aimed to comprehensively assess the acute toxicity of a well-characterized GO suspension to *Daphnia magna*. Conventional ecotoxicological endpoints (lethality, immobilization) and more sensitive, sublethal endpoints (heartbeat rate, feeding activity, and reactive oxygen species (ROS)) production were used. The possible normalization of the heartbeat rate and feeding activity in clean test medium was also investigated. The fate, time-dependent, and concentration-dependent aggregation behaviour of GO was followed by dynamic light scattering, UV-Vis spectroscopy, and zeta potential measurement methods. The EC_20_ value for immobilization was 50 mg/L, while, for physiological and behavioural endpoints, it ranged from 8.1 mg/L (feeding activity) to 14.8 mg/L (immobilization). The most sensitive endpoint was the ROS production with EC_20_ = 4.78 mg/L. 24-h recovery experiments revealed that feeding activity was restored only up to a certain level at higher concentrations, indicating that the potential environmental health effects of GO cannot be neglected. Alterations of normal physiology (heart rate) and feeding activity may be associated with increased risk of predation and reproductive decline, highlighting that GO may have impacts on population and food web dynamics in aquatic ecosystems.

## 1. Introduction

The growing use of carbon-based nanomaterials (CNMs) and their mass production have raised questions about their safety and environmental impact. However, data are still fragmentary. Toxicity characterization is still at an early stage and subject to criticism. In recent years, investigation of the potential adverse effects of an important CNM, graphene, on the aquatic ecosystem has attracted great attention, considering the likeliness of its release into the environment at significant levels due to its extensive production and use [1,2]. One of the most widely used graphene-family nanomaterial derivative is graphene oxide (GO), which is prepared by oxidative exfoliation of graphite, containing functional groups such as hydroxyl, carboxyl, and epoxy groups [3,4]. Graphene oxide may be introduced into the environment through its application as adsorbent for wastewater and drinking water treatment, material for solid-phase extraction, membrane for desalination, catalyst for aqueous organic pollutant oxidation and degradation, and coating material for filtration or even during the waste disposal of GO-containing products [5].

Graphene sheets with carboxyl, hydroxyl, and epoxy groups on the surface are able to form relatively stable suspensions [6]. However, upon release into aquatic environments, they are likely to interact with natural organic matter, inorganic ions, colloidal particles, or biocolloids. Therefore, their dispersion/aggregation behaviour can be very complex and altered upon interactions with natural system constituents modifying suspension stability that is governing the transport and fate of GO [6].

Zhao et al. [6] in their critical review about graphene in the aquatic environment highlighted that considerable challenges limit the understanding of the environmental fate, exposure, and risk of graphene-family nanomaterials. One of these challenges was identified to be the structure and surface properties of GO, such as the lateral size, C/O ratio, and the structural defects, which vary considerably depending on the applied synthesis routes, assisted processes, and original graphite materials inducing the different colloidal behaviours, adsorption capabilities, and toxicities of GO.

*Daphnia magna* is a filter-feeding zooplankton and a frequently used ecotoxicology test organism, representing an important trophic level in the food chain of aquatic ecosystems between algae and fish [7]. The toxicity of pristine graphene oxide and its functionalized derivatives to aquatic organisms is widely assessed, especially its effects on *D. magna* are investigated. However, there are further questions to be answered and aspects to be investigated. The most comprehensive scientific publications about the effect of GO on *D. magna* from the past few years revealed that the investigation of well-characterized GO suspensions at molecular, physiological, and behavioural levels associated with *D. magna* was of primary concern [8,9,10,11,12,13].

Taking into consideration the existing knowledge gaps on the acute ecotoxicity of GO to the key-member of the aquatic trophic chain, *D. magna*, our research focused on three different, but related aspects:

(i) Characterization of the fate and physico-chemical properties of the original GO suspension as well as its concentration-dependent and time-dependent aggregation characteristics and stability in the applied test medium under environmentally-relevant circumstances, since the characteristics of GO and the aggregation processes may determine bioavailability, and, hence, the toxicity of GO.

(ii) Demonstration of the sensitivity and feasibility of behavioural and physiological sublethal endpoints compared to the conventionally applied standard lethality and immobilization of *D. magna* for the toxicity characterization of GO. To the best of our knowledge, the effect of GO on the heartbeat rate of *D. magna* has not been tested so far. Although some results are available on GO uptake and depuration by *D. magna*, these investigations focused on bioaccumulation, determining body burden and the rate of depuration. In our opinion, this valuable information should be complemented with feeding activity studies, with the hypothesis, that a certain rate of depuration does not necessarily mean equivalently restored feeding activity, even if there is an implicit connection between these two phenomena. The extent of normalised physiology (e.g., heartbeat rate) and restored feeding ability are also scarcely investigated in the recovery studies.

(iii) Application of a comprehensive approach, which is a novelty. We applied various bioassay endpoints including the conventional, standardized ones (lethality and immobilization) and the sublethal ecotoxicity endpoints (heartbeat rate, feeding activity, and reactive oxygen species (ROS) production assay) complemented with recovery studies at different exposure durations to evaluate the toxic effect of GO on *Daphnia magna* and the involved toxicity mechanisms. We hypothesised that, besides immobility and lethality, the heartbeat rate and feeding activity and the physiological reactions and oxidative stress responses would be also significantly influenced by GO. Recovery studies in GO-free test medium were also carried out for different recovery periods, hypothesising the possible normalization of physiology and behaviour.

In summary, in order to bridge knowledge gaps, this paper is a contribution to the general effort toward a better understanding of the time-dependent and concentration-dependent ecotoxicity of a well-characterized GO suspension on the crustacean, *Daphnia magna*, using a comprehensive approach including both conventional, standardized, and more sensitive, sublethal ecotoxicity endpoints complemented with recovery studies. In line with the current research directions, our study is focused also on the fate and physico-chemical characterization of the original GO suspension as well as on its concentration-dependent and time-dependent aggregation characteristics and stability in the applied test medium since the characteristics of GO and the aggregation processes may determine bioavailability. Hence, the toxicity of GO.

## 2. Materials and Methods

### 2.1. Synthesis and Characterization of the GO Nanoparticles

Graphene oxide (GO) was obtained from natural graphite (Týn nad Vltavou, Czech Republic, average particle size 63 µm) by the improved Hummers’ method with a yield of 33% [14]. The pristine GO suspension was purified by successive centrifugation (Jouan BR4i Multifunction Centrifuge, Thermo Scientific, Waltham, MA, USA, 9000 min^−1^) and thorough washing with 1 M HCl and doubly distilled water until neutral pH was achieved. The stock suspension contained ca. 1 w/w% GO. A freeze-dried sample was prepared from this suspension for further characterization. The C/O ratio and the apparent surface area measured on the freeze-dried GO monolith were 2.6 and 20 m^2^/g, respectively. TG/MS (Thermogravimetry-Mass Spectrometry) results showed that this GO was thermally stable below 200 °C. The Hummers’ exfoliation significantly damaged the structure of the poly-condensed aromatic graphene layer. The intensity ratio of the characteristic G (graphitic) and D (defect) peaks, I_G_/I_D_ in the Raman dropped from 6.0 ± 0.5 in the pristine graphite to 1.18 ± 0.01 (Figure S1). For the ecotoxicity tests, a 100 mg/L GO stock suspension was prepared with distilled water. The concentration of this stock suspension was checked with UV-Vis spectroscopy [15].

### 2.2. Physico-Chemical Characterization of the Assembled Ecotoxicity Test Systems

#### 2.2.1. Electric Conductivity and pH Measurements

The pH of the pristine GO suspension and the assembled test systems containing test individuals were determined at the beginning of the experiments and after 24 and 48 h exposure in three parallels using a WTW (Wissenschaftlich Technische Werkstätten GmbH, Weilheim in Oberbayern, Germany) instrument containing a pH 330 instrument and a pH electrode (Electrode Sentix 81). Electric conductivity was determined with a Consort C535 instrument (Turnhout, Belgium).

#### 2.2.2. Dynamic Light Scattering Method Characterization and Zeta Potential Determination

The hydrodynamic diameter (HDD) and polydispersity index (PDI) of GO particles and their agglomerates in the assembled test systems were determined at 25 °C by using a dynamic light scattering device (Brookhaven DLS Instruments, Holtsville, NY, USA) that was operated with an Ar laser light (Coherent Innova 70C Argon laser) at a wavelength of 488 nm and a power of 30.0 mW and using a Brookhaven Instruments Corporation BI-DS1 detector. Light scattering was detected at an angle of 90°, pinhole: 100 μm measurement time: 2 min, and measurement interval for correlation times: 2 μs–100 ms. Autocorrelation functions were evaluated by the second order cumulant model to yield average HDD and PDI. Three replicate measurements were made at zero time points and at the end of the applied exposure times of the applied ecotoxicity assays accordingly. The zeta potential of GO particles in the test medium was measured at 25 °C with Brookhaven ZETAPALS Instrument (Brookhaven Instrument Corp., Holtsville, NY, USA).

The electrophoretic mobility of GO particles was measured in three replicate measurements. Then the electrophoretic mobility values were converted to zeta potential by the Henry equation with the Smoluchowski approximation.

#### 2.2.3. UV-Vis Spectroscopy for GO Concentration Determination

Optical absorbance measurements were performed with a Specord 200 (AnalytikJena, Jena, Germany) UV-Vis Spectrophotometer. Data were collected in the whole UV-Vis range (190–1100 nm). The typical UV absorption peak of the GO appears around 230 nm.

### 2.3. Applied Ecotoxicity Test Methods

#### 2.3.1. *Daphnia magna* Cultures

A colony of *Daphnia magna* cultured in the laboratory was used in a series of experiments. The test animals were cultured in 5 L beakers in a 21.5 ± 1 °C thermostatic chamber with 16:8 h light:dark cycle (illumination: Juwel Aquarium, Day-Lite, 15 W, 438 mm lamp, 560 Lumen, 6500 K) and fed everyday by 2 mL of alga suspension of 10^9^ cell/mL concentration cultivated in the laboratory containing *Chlorella sorokiniana*. For maintaining *D. magna,* natural surface water (Collection site: Lake Balaton, Alsóörs, Hungary; 46°97′ N, 17°95′ E; pH = 8.65, EC = 620 µS) was used. To check the sensitivity of the *D. magna* culture, acute toxicity tests were performed with potassium dichromate (K_2_Cr_2_O_7_) as a reference toxicant at about every six-month interval. Sensitivity of *D. magna* culture to K_2_Cr_2_O_7_ ranged within the limits (EC_50_, 24 h = 0.6–2.1 mg/L) set by guideline OECD 202 [16].

#### 2.3.2. *Daphnia magna* Lethality and Immobilization Assay

The *Daphnia magna* acute lethality and immobilization tests were performed based on the OECD 202 [15] test protocol.

#### 2.3.3. *Daphnia magna* Heartbeat Rate Assay

Non-pregnant, 10-day-old *D. magna* individuals (not from the first brood) were used for the test. The animals were not fed during the test. The dissolved O_2_ concentration was more than 3 mg/L at the end of the test, as recommended by the OECD 202 Guideline [16]. The applied nominal concentrations of the tested GO suspensions were 3.125, 6.25, 12.5, 25, and 50 mg/L. With the help of a special fabric spoon, 5 animals were placed into four parallels of 25-mL test solution in 50-mL test vessels. Distilled water was applied as a control, containing the growth medium in the same proportion as the GO samples. The beakers were covered with a translucent plastic film to avoid evaporation and concentration of the test suspension during the experiment. These experimental vessels were incubated under the same conditions as described in Section 2.3.1. The heartbeat rate of the animal was measured under a stereomicroscope (NIKON SMZ800 twice during the test, after 24-h and 48-h exposure times). The test animals were placed onto a single cavity microscope slide in a 50-μL droplet of the test suspension, where the heartbeat rate of the test animals was measured one-by-one (individually), three times for 10 s. Each measurement was repeated three times [17].

#### 2.3.4. *Daphnia magna* Feeding Activity Assay

The test was carried out based on Kamaya et al.’s [18] method with major modifications. For the test, non-pregnant 10-day-old *D. magna* individuals were used, which were not derived from the first brood and were starved for 3 days prior to the test and were not fed either during the test. The dissolved O_2_ concentration (determined with WTW ‘Portable Meter 340i’) was more than 3 mg/L at the end of the test as recommended by the OECD 202 Guideline [16]. The applied nominal concentrations of the tested GO suspensions were 3.125, 6.25, 12.5, 25, and 50 mg/L. Five animals were placed into four parallels of 25-mL test solution in a 50-mL test vessel with the help of a special fabric spoon. Distilled water was applied as a control with growth medium in the same proportion as in the GO-containing samples. The incubation was carried out under the same conditions as described in Section 2.3.1.

After 48 h of exposure time, the Daphnia individuals from two test vessels were combined and the ten individuals were transferred into 10-mL of a 3-µL/mL concentration fluorescent microsphere suspension (Life Technologies, FluoSpheres™ Carboxylate-Modified Microspheres, 0.2 µm, yellow-green fluorescent (505/515), 2% solids) diluted with the original *D. magna* growth medium. The individuals were kept in the fluorescent microsphere suspension and fed for 20 min. Then they were taken out with a special fabric spoon, and washed thoroughly with distilled water in order to remove the microspheres adhered onto their carapace and appendages, which could influence the results. The washed individuals were transferred into a micro test tube in 1 mL of distilled water, and then homogenized for 30 s with UP 200H Ultrasonic Processor (Hielscher Ultrasonics GmbH, Teltow, Germany) with the following settings: cycle: 1, amplitude %: 82. The homogenized Daphnia-microsphere suspensions were pipetted into three parallel wells of a 96-well microtiter plate and the fluorescence intensity of the wells was measured by FLUOstar Optima BMG Labtech microplate reader using the excitation wavelength of 485 nm and the emission wavelength of 520 nm.

Additionally, feeding activity experiments were visualized using fluorescence microscopy. After incubation with fluorescent microsphere suspension for 20 min and washing twice with distilled water, the treated *D. magna* individuals were placed onto microscope slides and examined by Nikon ECLIPSE E400 fluorescence microscope, under 40× magnification, using a Nikon CFI Plan Achromat 4× objective lens (Nikon Corporation, Tokyo, Japan).

#### 2.3.5. Oxidative Stress Assay

ROS production was determined based on the method of Ulm et al. [19] with minor modifications using 2′,7′-dichlorodihydrofluorescein diacetate (DCFH-DA), which is nonfluorescent unless oxidized by intracellular ROS. The applied nominal concentrations of the tested GO suspensions were 3.125, 6.25, 12.5, 25, and 50 mg/L. After 24 and 48 h of exposure periods, ten *D. magna* individuals in three parallels were transferred to borosilicate glass vessel (with a diameter of 2 cm, height: 1 cm), each test vessel containing approximately 10 mL of 50 mM phosphate buffer (PB pH 7.4). *D. magna* individuals were washed three times with PB pH 7.4 prior to incubation with DCFH-DA to minimize interferences of graphene oxide particles with fluorescence measurements. Then, *D. magna* individuals were incubated with 10 μM DCFH-DA for 30 min at 37 °C. They were then washed twice with PB pH 7.4. These ten individuals were homogenized for 30 s with UP 200H Ultrasonic Processor (Hielscher Ultrasonics GmbH, Teltow, Germany) with the following settings: cycle: 1, amplitude %: 82. The homogenized Daphnia suspensions were pipetted into three parallel wells of a 96-well microtiter plate and the fluorescence intensity of the wells was measured by a FLUOstar Optima BMG Labtech microplate reader using the excitation wavelength of 485 nm and the emission wavelength of 520 nm. The data are expressed as a percentage fluorescence when compared with the relevant negative controls.

#### 2.3.6. Recovery after GO Exposure

In the case of *D. magna* feeding activity and heartbeat rate, recovery studies were carried out by placing the test individuals into pure Daphnia test medium for 4-h and 24-h recovery periods after 24-h and 48-h exposure to GO at different concentrations. The aim of these studies was to investigate whether normal physiology and behavior can be restored. After the recovery period, the heartbeat rate and feeding activity were determined according to the methods described in Section 2.3.3 and Section 2.3.4.

### 2.4. Data Evaluation and Statistical Analysis

In the case of each ecotoxicological endpoint, inhibition percentage values (H%) were calculated when compared to the control. One-way analysis of variance (ANOVA) was performed by STATISTICA 13^®^ software (version 13.0, StatSoft. Inc., Tulsa, OK, USA), identifying significant effects (*p* < 0.05). The homogeneity of variances was examined with Leven’s test. Fischer’s least significant difference test was carried out to compare the effects of the different GO concentrations. In case of significance, the Lowest Observed Effect Concentration (LOEC) values were determined using Dunnett’s test (α = 0.05). Effective concentration (EC_20,_ EC_50_) values were determined using OriginLab 8.0 software with a logistic function fitting (y = A_2_ + (A_1_ − A_2_)/(1 + (x/x_0_)^p^))).

## 3. Results

### 3.1. Results of the Physico-Chemical Characterization of the Assembled Ecotoxicity Test Systems

Since the exposure systems are identical in the case of the different ecotoxicity endpoints, data were collected from each set of experiment and average pH and EC values were calculated for all experiments. The pH of the assembled test systems fell within the range of 7.75–8.21 and no significant change compared to the control was experienced (Table S1). The electric conductivity of the control sample (EC (0 h) = 583 ± 8 µS/cm) was significantly different from the test systems containing GO (EC (0 h) = 476 ± 15 − 493 ± 8 µS/cm). However, there was no significant change between the EC of samples with different GO concentrations (*p* < 0.05) (Table S1 of the Supplementary material section).

After 24 h of exposure, a thin layer of clean supernatant and a thick layer of sedimented GO suspension with aggregated particles could be examined. These upper and lower phases were separately sampled and submitted for zeta potential and DLS measurements. The original distilled water diluted GO stock suspension and can be considered stable based on its zeta potential values: −40.4 ± 1.4 mV. The zeta potential values shown in Table 1 fell within the range from −10.2 +/− −8.2 to −18.9 +/− −2.9 mV. These results suggest that zeta potential values at all GO concentrations, set by diluting the stock GO suspension with *D. magna* culture medium, are far from the stability limit value.

**Figure 1:**
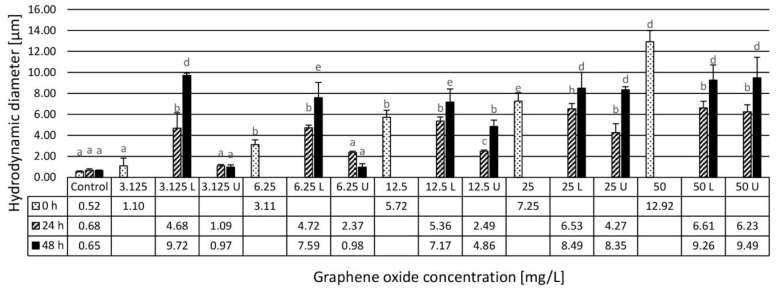
Mean values of hydrodynamic diameter (in nm) of GO particles in the assembled test systems at a zero time point and after 24 and 48 h of exposure reported from *n* = 3 determinations per sample. U: upper phase, L: lower phase. Letters on the columns indicate significant differences (level of significance: *p* < 0.05).

**Figure 2:**
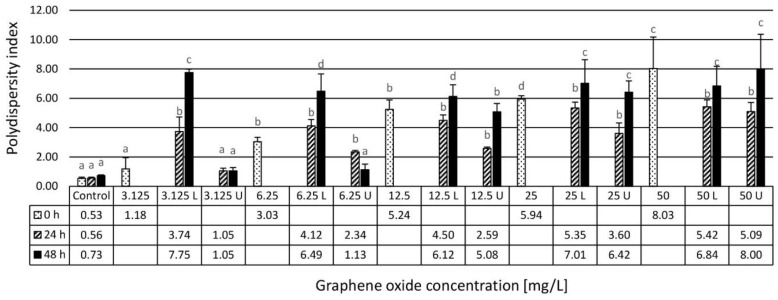
Mean values of polydispersity index (PDI) of GO particles in the assembled test systems after 24-h and 48-h of exposure reported from *n* = 3 determinations per sample. U: upper phase, L: lower phase. Letters on the columns indicate significant differences (level of significance: *p* < 0.05).

At the beginning of the experiments, control samples were analyzed and a submicron hydrodynamic diameter (HDD) was obtained with a relatively narrow PDI. This suggests the absence of larger, micron-sized particles in the initial system. The addition of GO particles significantly increased the average HDD as well as the PDI values. At all concentrations, the average diameter increased to above one micron with a general increase at incremental concentrations, particularly after 24 h. There is a clear difference between the upper and the lower phases at concentrations below 50 mg/L with a significantly larger particle size in the lower phase within the size range of a few microns up to around 7 μm. The upper phases were apparently transparent showing diameters ranging from 1 to 4 μm. PDI values also increased in general with concentration, with very broad size distributions in the lower phase indicating a strong aggregation tendency. PDI of the upper phases was generally lower at the lowest GO concentration approaching to that of the control samples. The aggregated particles accumulated mostly in the lower phase except for the highest GO concentration (Figure 1 and Figure 2).

Due to the strong instability of GO in the applied test medium, GO floccules were formed almost immediately after diluting the original stock GO suspension with *D. magna* test medium, which led to a slow phase separation-like behavior. Constant agitation of the test medium was not applied to avoid disturbance of normal behavioural patterns since *D. magna* is abundant in lakes and still-water ponds and agitation of the test medium may result in altered behaviour even in an uncontaminated test system. Considering the aggregation and sedimentation behaviour of GO in natural surface waters, it is recommended to test the effect of GO under environmentally relevant conditions, which also lack artificial stirring or agitation. By the time of the 24-h sampling, a clear upper aqueous phase was formed in the test vessels, which was also confirmed by several physico-chemical methods such as UV-Vis spectroscopy, particle size, and electophoretic mobility measurements and scanning electron microscopy. The volume of this upper and lower phase shows the systematic influence of the initial GO concentration. The exact parameters of the GO-depleted upper and the GO-rich lower phases (volume, depth) in the cylindrical test vessels with a diameter of 40 mm were recorded and reported in Table 2. The fate of the GO particles was followed in both phases by UV-Vis spectroscopy at time zero as well as after 24 h and 48 h of exposure. Taking into account the living nature and, thus, the continuously changing background of the test systems, no attempt was made to determine the exact GO concentration in the two phases. Representative sampling was particularly challenging in the inhomogeneous lower phase. Nevertheless, a clear tendency of GO sedimentation over time was confirmed by the UV-Vis spectra. UV-Vis spectra of the measured samples revealing the influence of the GO concentration are shown in Figure S2 of the Supplementary material section.

### 3.2. Results of the Ecotoxicity Experiments

Based on the results of the conventional, standardized lethality and immobilization assays, after 24 h of exposure, there was no sign of any lethal or immobilization effect. After 48 h of exposure, the most concentrated GO suspension significantly affected survival and swimming ability of *D. magna*, 20 ± 7% lethality and 10 ± 5% immobilization effect was caused by 50 mg/L GO (Figure S2 of the Supplementary material section).

**Figure 3:**
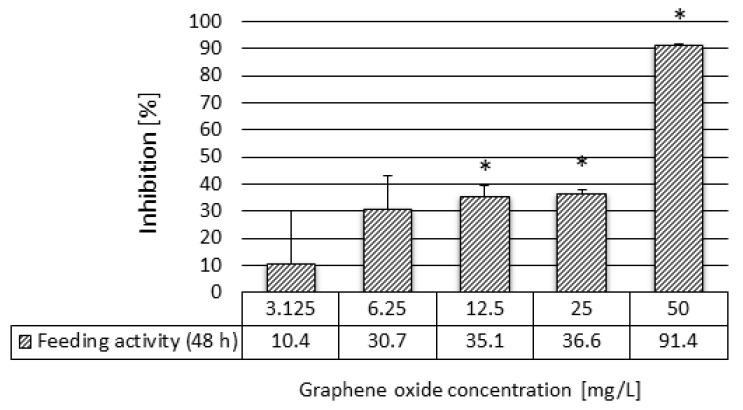
The effect of graphene oxide on the feeding activity of *D. magna* after 48 h of exposure. Significant effect compared to the control is marked by an asterisk (*).

**Figure 4:**
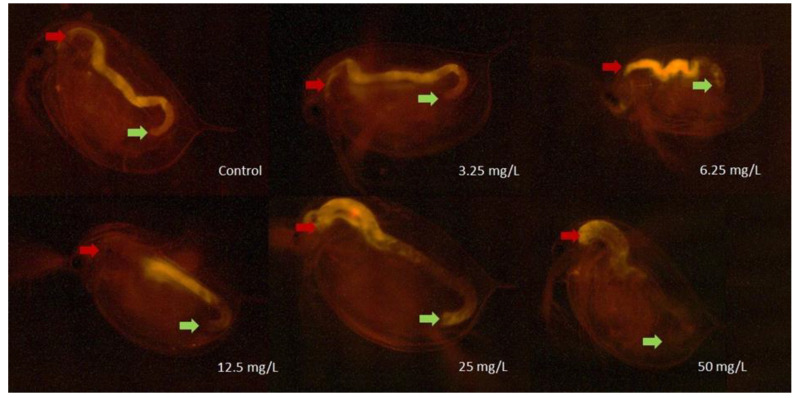
Images of *D. magna* individuals after 48 h of exposure to different concentrations of GO and after feeding on fluorescent microbeads visualized by fluorescence microscopy (Nikon ECLIPSE E400 fluorescence microscope, 40× magnification, Nikon CFI Plan Achromat 4× objective lens). The beginning of the digestive tract is marked by red arrows, while the end is marked by a green arrow.

Furthermore, 48 h of exposure to GO in the 3.125–50 mg/L concentration range resulted in a severe decrease of the feeding activity. In addition, 50 mg/L GO decreased the feeding activity of *D. magna* by ~91%. In the 6.25–25 mg/L concentration range, the inhibitory effect was 30.7–36.6%, which proved to be significant in the test systems containing 12.5 and 25 mg/L GO. The inhibitory effect within this concentration range did not show concentration-dependence (Figure 3). The uptake of fluorescent microbeads was visualized by a fluorescence microscopy technique. The quantified uptake of fluorescent beads by daphnids (Figure 3) was proportional with the length and colour intensity of the area filled with fluorescent microbeads in the digestive tract of daphnids, as shown in the microscopic images of Daphnia individuals from different treatments (Figure 4).

**Figure 5:**
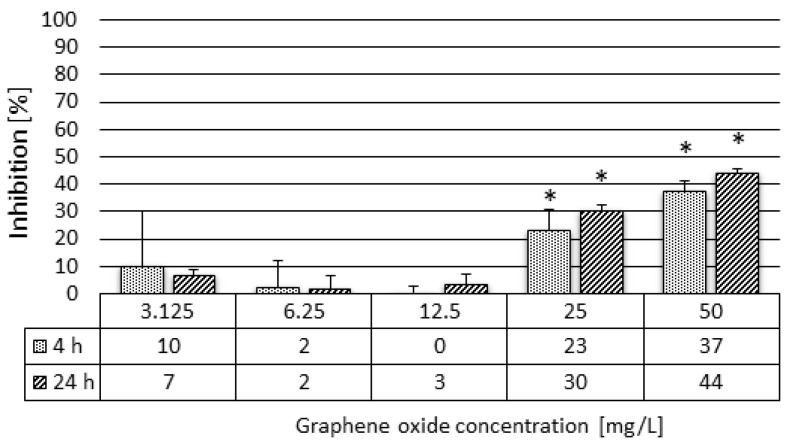
Inhibition of feeding activity of *D. magna* exposed to GO for 48 h after 4 h and 24 h of a recovery period in clean growth medium. Significant effect when compared to the control is marked by an asterisk (*).

In the second phase of the feeding activity assays with *D. magna* after 48 h of exposure to different concentrations of GO (3.125–50 mg/L), the potential recovery of the feeding rate was tested by replacing the GO-exposed Daphnia individuals into a clean Daphnia growth medium for 4 h and 24 h. Results showed that, after 4 h of a recovery period in the clean growth medium, the feeding activity was restored to a normal rate of feeding when compared to a control at 3.125, 6.25, and 12.5 mg/L concentrations (Figure 5). After 4 h of a recovery period in the two most concentrated GO suspensions (25 and 50 mg/L), the inhibitory effect was still 23% and 37%, respectively. At 25 and 50 mg/L GO concentrations, the extension of the recovery period did not result in the intensification of the feeding activity compared to the levels detected after only 4 h of the recovery period, but, rather, in the increase of the inhibitory effect by 7%, this increase was not statistically significant.

**Figure 6:**
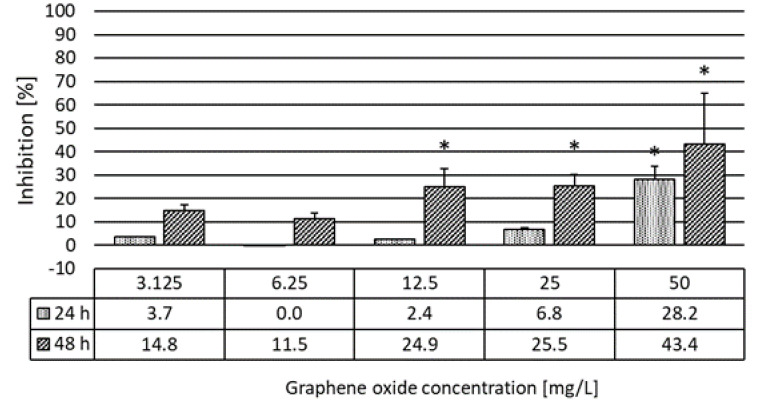
The effect of graphene oxide on the heartbeat rate of *D. magna* after 24 and 48 h of exposure. Significant effect compared to control is marked by asterisk (*).

**Figure 7:**
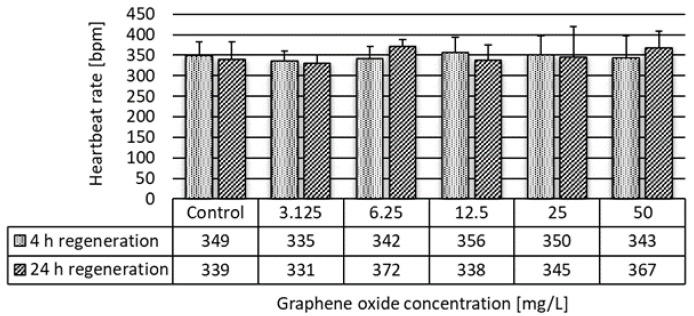
Heartbeat rate of *D. magna* exposed to GO for 48 h after 4 h and 24 h of a recovery period in clean growth medium.

Inhibition of the heartbeat rate showed a growing trend with both increasing concentration and exposure time. After 24 h of exposure, the heartbeat rate significantly (H% = 28%) decreased in the most concentrated GO suspension. After 48 h of exposure, the heartbeat rate significantly decreased at 12.5 and 25 mg/L concentrations (Figure 6) as well. After exposure to GO for 48 h, recovery experiments were carried out as in the case of feeding activity experiments. We found that, after replacement of the daphnids into clean growth medium for 4 h, the heartbeat rate was normalized and no significant difference compared to the heartbeat rate of the control group was determined (Figure 7).

The fluorescence intensity of the oxidized 2′,7′-dichlorodihydrofluorescein diacetate (DCFH-DA) is directly proportional to the intracellular ROS level. The effect of graphene oxide on reactive oxygen species (ROS) production in *D. magna* after 48 h of exposure exhibited a direct relationship between the applied GO concentration and the relative ROS generation when compared to the control.

**Figure 8:**
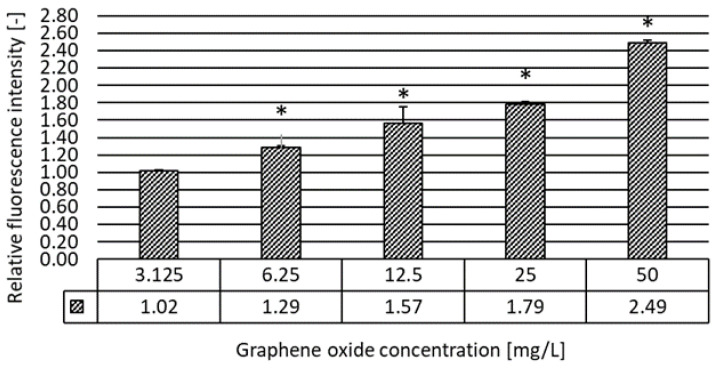
The effect of graphene oxide on ROS production in *D. magna* after 48 h of exposure in relative fluorescence intensity units when compared to the untreated control. Significant effect compared to the control is marked by an asterisk (*).

The results illustrate a significant increase in ROS levels after treatment with GO at all tested concentrations except for the lowest 3.125 mg/L (Figure 8). The intensity of the relative fluorescence was the highest at 50 mg/L GO concentration, which is ~2.5 times higher than in the control.

## 4. Discussion

### 4.1. Physico-Chemical Characterization and Stability Evaluation of Graphene Oxide

The fate and aggregation behavior of GO was characterized by DLS, electrophoretic mobility determination, and UV-Vis spectroscopy methods in the assembled test systems. About the stability of NPs, the zeta potential can give an idea. The zeta potential value represents the magnitude of the electrostatic or charge attraction between particles. Particles with a zeta potential below −30 and over +30 mV are considered stable (at pH 7), while values between −30 and +30 mV NPs are not considered stable. Instead, they are expected to aggregate over time [20]. The zeta potential values measured in the assembled test systems indicate that GO suspensions are outside the stability limit and tend to form aggregates at all concentrations in which the phenomenon could be easily examined by bare eyes over time. Due to the strong instability of GO in the applied test medium, GO floccules were formed, which led to a slow phase separation-like behavior and daphnids tended to avoid the GO-enriched lower phase. However, the volume of this lower phase was different at each applied concentration and showed the systematic influence of the initial GO concentration and time as confirmed by Reference [21] when diluting GO suspension with tap water. Due to GO flocculation and sedimentation representative sampling becoming particularly challenging in the inhomogeneous lower phase, no attempt was made to determine the GO concentration in the two phases. Nevertheless, a clear tendency of GO sedimentation over time was confirmed by the UV-Vis spectra as well. The above described phenomenon was also reported by Lv et al. [21] when testing GO, highlighting that the presence of *D. magna* resulted in enhanced aggregation of GO when compared to abiotic circumstances.

The magnitude of the average hydrodynamic diameter (HDD) did not correlate strongly with the concentration of GO after 48 h in the upper phase. It was similar (~1 µm) at 3.125 and 6.25 mg/L, indicating the presence of aggregated micron-size particles even at low GO concentrations. Aggregation and a change in GO characteristics such as HDD may be influenced by several parameters such as pH, ionic strength, and natural organic matter might affect its bioavailability and, hence, play an important role in determining toxicity [5,22].

The interaction of nanoparticles with biological systems is widely studied by colloid chemical methods, i.e., the size change (aggregation) of particles by dynamic light scattering (DLS) and the change of surface charge, and, usually, as a consequence, the stability by zeta potential measurements. DLS can be used for the characterization of biomolecules, nanoparticles, and their aggregation [23]. However, it is not selective. Thus, all components in a complex biological system might contribute to the signal. Considerable work was directed to improve the selectivity of the method, e.g., by combining fluorescent marking with DLS or using depolarization techniques [24]. Similarly to DLS, it is difficult to get accurate values for zeta potential in complex systems since zeta potential is affected by various factors including the properties of particles (surface functionalization, diameter), particle concentration, particle-particle interactions, buffer components, the presence of proteins, or other biomolecules, etc. A very comprehensive review on the topic was published by Lowry et al. [25]. By limiting the measurement time, the effects of particle aggregation and blackening (screening) the electrode by macromolecular or other coating of the electrode can be minimised. Using a low electric field can help avoid the change of the sample during the measurement. When keeping all limitations in mind, it is generally useful to compare the zeta potential with the rule-of-thumb stability limit (30 mV), but the possibly high standard error of zeta potential values must be taken into account.

### 4.2. Acute Ecotoxicity of Graphene Oxide to Daphnia Magna

The present study clearly displayed the potential of GO to affect *Daphnia magna* physiology, behaviour, and oxidative stress reactions, whereas the extent of the effects depend on the GO concentration, exposure period, and tested endpoints besides the physico-chemical parameters.

Similarly to the results of our conventional, standardized lethality, and immobilization assays, Lv et al. [21] also described non-severe acute toxicities, including immobility (EC_50_ (72 h) = 44.3 mg/L) and mortality of *D. magna* (EC_50_ (72 h) = 45.4 mg/L) upon GO. These results are also in accordance with Zhang et al.’s [26] findings reporting EC_50_ (48 h) = 84.2 mg/L and mitigation by 32.3% of the acute toxicity in the presence of humic acid (25 mg/L). The effective concentration values determined based on our results are not directly comparable with those determined by Lv. et al. [21] since the maximum exposure period in the case of our experiments was 48 h. However, it could be stated that the EC_20_ (48 h) = 50 mg/L for mortality was close to their results.

The GO-mediated feeding behaviour showed a strong relation with the polydispersity index (PDI) and the hydrodynamic diameter (HDD) determined in the test medium at different GO concentrations. There was no significant inhibition at the lowest GO concentrations (3.125 and 6.25 mg/L), while the average HDD was much lower (0.97–0.98 µm) than at the next, higher concentrations tested at which the PDI and the HDD increased exponentially. At these higher PDI and HDD values, the feeding inhibition became significantly elevated, indicating that the toxicity was closely related to these parameters. The elevated toxicity may be due to the higher particle size of GO at 12.5–50 mg/L (4.86–9.49 µm), which could be retained by the filtering apparatus of daphnids, while the accumulation level of the smaller particles was found to be very low [19].

Based on previously reported depuration studies at lower GO (<1 mg/L) concentrations, achieving almost complete depuration of GO [27], our aim was to study the effect of the maximum GO concentration in the test medium, which would still induce a satisfactorily restorable adverse effect on feeding. Our results revealed altered feeding behaviours after 24 and 48 h of GO exposure, which could be recovered only to a certain extent even after 24 h of a recovery period in a clean test medium. Guo et al. [27] measured the rate of GO depuration after the exposure of daphnids to GO at 50, 100, and 250 µg/L for 24 h, and then replaced them into clean artificial freshwater for 1, 4, 10, and 24 h. GO uptake was expressed in body burden units (µg graphene/mg Daphnia dry mass) before and after depuration. The results showed that Daphnia excreted 46% and 64% of the accumulated graphene from their guts after being exposed to a graphene concentration of 100 and 250 μg/L, respectively, in clean artificial freshwater (AF), resulting in roughly constant body burden after elimination for 24 h in clean AF for Daphnia exposed to different concentrations during the uptake experiments. In addition, if Daphnia were fed algae during the depuration period, the body burdens decreased more than 90% during the first 4 h.

Furthermore, the presence of 10 mg/L humic acid (HA) in the AF also increased the graphene excretion rate compared to the clean AF condition. It was supposed that this enhancement may be attributed to the interactions between HA and graphene or to the similarities between the molecule size of HA and algae, but further studies are necessary to understand the mechanism [27].

Assuming a direct link between the GO depuration rate and restored feeding activity, it could be stated that, in our experiments, roughly a complete GO depuration could be reached even after 4 h in clean growth medium in the case of exposure to a lower GO concentration of up to 12.5 mg/L for 48 h, according to the restored feeding activity, compared to the control, without addition of algae or of any kind of natural organic matter, which proved to facilitate GO depuration from the gastric tract, according to Guo et al. [27]. Based on these results, acute exposure to GO at a relatively high concentration (12.5 mg/L) did not result in irreversible damage in the normal food uptake rate. However, the effect of chronic exposure to GO at a low and a very low (ng–µg/L) level is still an open question to be investigated as well as the potential risks of other contaminants, such as organic pollutants and heavy metals present in the aquatic ecosystems, which may adsorb onto GO nanoparticles [28,29].

However, Loureiro et al. [30] investigated the effect of pegylated GO (nGO-PEG) nanoparticle suspension at 2–400 µg/L in 21 d chronic toxicity test, following reproduction rate and body length, which were strongly connected to the normal rate of feeding, revealing that body length was not affected at any of the applied nGO-PEG concentrations. However, all tested concentrations of nGO-PEG resulted in a significant increase in the reproductive output. These findings support the importance of the ecotoxicity testing of different GO particles with and without further modifications, altering bioavailability and resulting in different toxicity patterns, even eco-friendly forms of functionalized GO.

Based on our knowledge, the effect of GO on the heartbeat rate of *D. magna* has not been investigated yet. However, it could be hypothesized that this physiological endpoint may be altered by previously reported GO induced oxidative stress [12]. According to our results, the heartbeat rate was found to be lowered due to GO exposure for 48 h at 12.5, 25, and 50 mg/L concentrations. However, it was also found that, after replacement of the daphnids into clean growth medium for 4 h, the heartbeat rate was normalized and no significant difference compared to the control group was determined. In the current literature, there is only one example of the testing of CNM with a *D. magna* heartbeat rate assay. Lovern et al. [31] investigated the effect of nano-C_60_ and a fullerene derivative, exposing *D. magna* to 2 ppm C_60_H_x_C_70_H_x_ for 1 h. They found that the nano-C_60_ was the only suspension to cause a significant change in the heartbeat rate when compared to the control, increasing the average rate from 43.6 bpm to 317.09 ± 7.21 bpm after exposure. This suggests that the functional groups may have a drastic effect on the sublethal effects (such as behavior and physiology) of nanoparticles.

The key potential mechanism of nanoparticles’ toxicity is supposed to be via oxidative stress with reactive oxygen species [32]. Although the oxidative stress inducing property of GO at certain concentrations in different aquatic organisms [33,34] and in *D. magna* [21] was confirmed, our aim was to determine the extent of GO-induced oxidative stress within the concentration range used in our experiments as well as to complement our ecotoxicity toolkit with a method having the ability to reveal the potential underlying molecular mechanisms potentially influencing the physiological and behavioral endpoints. We determined a concentration-dependent significant increase of the oxidative stress level within the 6.25–50 g/L GO concentration range. Zhang et al. [26] found oxidative damage caused by GO in *D. magna,* while resulting approximately 135%, 130%, and 250% increase in ROS, superoxide dismutase (SOD), and catalase (CAT) activity, respectively, at 10 mg/L GO concentration in the acute test. A significant increase in ROS generation was confirmed by Cano et al. [12] when exposing *D. magna* to GO even at a very low concentration (0.1 mg/L) for 72 h.

### 4.3. Evaluation of the Sensitivity of the Applied Ecotoxicity Endpoints

To be able to determine effective concentration values enabling the comparison of standard and innovative endpoint sensitivity, relatively high GO concentrations were applied (3.125–50 mg/L). The effective concentration values causing 20% and 50% inhibition were determined in the case of all ecotoxicity assays with *D. magna* (Table 3). The lowest observed effect concentration values were also determined by identifying significant effects (*p* < 0.05) in the tested GO concentration range. As could be expected, the conventional, standardized lethality and immobilization assays were the less sensitive among all ecotoxicity assays with *D. magna*. The innovative, sublethal ecotoxicity endpoints showed higher sensitivity, similarly to Bownik [35], which also confirmed the importance of refined, more sensitive sublethal endpoints in nano-ecotoxicology. The most sensitive assay was the determination of oxidative stress with the lowest EC (Effective Concentration) and LOEC (Lowest Observed Effect Concentration) values. Feeding activity also proved to be sensitive. However, in this case, EC and LOEC values were approximately two times higher than those of the oxidative stress assay. Based on the LOEC values, the heartbeat rate test was as sensitive as the feeding activity, but the GO concentration, resulting in 20% inhibition of the heartbeat rate, was higher than in the case of feeding activity. Based on these results, future experiments should address GO toxicity characterization at environmentally-relevant concentrations with these refined, more sensitive ecotoxicity endpoints in the case of chronic exposure, supplemented with recovery studies.

## 5. Conclusions

The results of the present study show alterations in a concentration-dependent manner in the heartbeat rate, feeding activity, and an increased ROS generation in *D. magna*, which is the most commonly used test organism in nanoecotoxicology, exposed to GO for 48 h. GO-induced significant inhibition of the heartbeat rate, which was normalized after 4 h of a recovery period in a clean test medium. The recovery of the feeding activity was also experienced up to a certain level. However, it was more dominant at lower GO concentrations. Our results clearly demonstrate that, in the aquatic environment, GO may result in adverse effects on water flea physiology, behavior, and biomarker responses. These harmful, altered individual responses may mediate subsequent ecological consequences. Physico-chemical measurements revealed the presence of large, micro-scale GO aggregates, particularly in the lower, sedimented phase with a considerable increase in the average hydrodynamic diameter over time. Although daphnids tended to avoid GO floccules and were mainly swimming in the GO-depleted upper phase, their digestive tract was filled with GO particles. Our results prove that the aggregation of GO nanoparticles in natural waters does not necessarily lower the inhibitory effect due to decreased bioavailability. The aggregated micro-size particles exert their toxic effect via uptake, getting stuck in the alimentary canal, affecting normal physiology and behavior physically and, at the molecular level, causing oxidative stress. The observed GO-mediated physiological alterations and antioxidation mechanism in *D. magna* after exposure to GO justify the need to examine these more sensitive sublethal responses besides standard test endpoints.

## Figures and Tables

**Table 1:** Zeta potential values measured in the assembled test systems after 24 and 48 h of exposure reported from *n* = 3 determinations per sample.

Graphene Oxide Concentration [mg/L]	Phase	Zeta Potential [mV]		
		0 h	24 h	48 h
**Control**	–	−14.9 +/− −9.2	−16.7 +/− −3.6	−14.9 +/− −5.8
3.125	Upper Lower	−18.4 +/− −3.2	−14.6 +/− −4.9	−10.2 +/− −8.2
			−17.0 +/− −3.4	−16.3 +/− −2.5
6.25	Upper Lower	−17.9 +/− −2.4	−14.8 +/− −3.4	−15.8 +/− −3.1
			−16.4 +/− −3.4	−14.0 +/− −3.1
12.5	Upper Lower	−18.9 +/− −2.9	−17.6 +/− −3.0	−12.2 +/− −5.3
			−13.6 +/− −3.1	−14.9 +/− −3.1
25	Upper Lower	−18.0 +/− −4.8	−15.1 +/− −2.9	−11.5 +/− −5.0
			−13.6 +/− −3.4	−13.9 +/− −3.0
50	Upper Lower	−12.2 +/− −4.8	−14.5 +/− −2.7	−16.4 +/− −2.8
			−13.4 +/− −7.3	−14.3 +/− −3.2

**Table 2:** Volume (mL) and depth (mm) of the clear, GO-depleted upper phase and the GO-rich lower phase in the assembled test systems after 48 h of an exposure period.

	Graphene Oxide Concentration [mg/L]											
	Control		3.125		6.25		12.5		25		50	
	Vol.	Depth	Vol.	Depth	Vol.	Depth	Vol.	Depth	Vol.	Depth	Vol.	Depth
Upper phase	–	–	19.8	16	18.4	15	16.3	13	13.9	11	11.8	9
Lower phase	–	–	5.2	4	6.6	5	8.8	7	11.1	9	13.2	11

**Table 3:** Effective concentration (EC) and lowest observed effect concentration (LOEC) values [mg/L] of GO for the applied ecotoxicity assays with *D. magna* after 24 and 48 h exposure.

Ecotoxicity Assay	Exposure Period	EC_20_	EC_50_	LOEC
Lethality	24 h	>50	>50	>50
	48 h	>50	>50	>50
Immobilization	24 h	>50	>50	>50
	48 h	50	>50	50
Heartbeat rate	24 h	41.84	>50	50.00
	48 h	14.77	>50	12.50
Feeding activity	48 h	8.10	29.18	12.50
Oxidative stress	48 h	4.78	12.55	6.25

## Supplementary Materials

The following are available online at https://www.mdpi.com/2079-4991/10/10/2048/s1. Table S1: Electric conductivity and pH values measured in the assembled test systems after 24 and 48 h of exposure reported from *n* = 3 determinations per sample. Significant differences when compared to the control are marked by bold italics at each time point. Figure S1. The fate of the GO particles in the test medium followed by UV-Vis spectrometry (a) in the sample with 25 ppm initial GO concentration, (b) in the lower (GO-rich) layers of the samples with a different initial GO concentration, and (c) in the GO depleted (upper) layers of the samples with different initial GO concentration. Figure S2. The effect of graphene oxide on the survival and swimming ability of *D. magna* after 48 h of exposure. Standard deviation values were less than 10% in all cases. Significant effect compared to control is marked by asterisk (*).
