# Supplementary material for: Ecotoxicity Assessment of Graphene Oxide by Daphnia magna through a Multimarker Approach from the Molecular to the Physiological Level including Behavioral Changes
Source: Nanomaterials (Basel). 2020 Oct 16;10(10):2048. doi: 10.3390/nano10102048 (PMC7603018; doi:10.3390/nano10102048)
Supplement: Supplementary file 1 [file nanomaterials-10-02048-s001.pdf]

Supplementary Materials

Ecotoxicity Assessment of Graphene Oxide by *Daphnia magna* through a Multimarker Approach from the Molecular to the Physiological Level including Behavioral Changes

Ildikó Fekete-Kertész [1], Krisztina László [2], Csilla Terebesi [1], Benjámin Sándor Gyarmati [3], Shereen Farah [2], Rita Márton [1] and Mónika Molnár [1*]

[1] Budapest University of Technology and Economics, Faculty of Chemical Technology and Biotechnology, Department of Applied Biotechnology and Food Science, Environmental Microbiology and Biotechnology Group, Műegyetem rkp. 3., Budapest, 1111 Hungary; fekete.kertesz.ildiko@mail.bme.hu (I.F.K.); terebesics@gmail.com (Cs.T.); ritamarton34@gmail.com (R.M.).

[2] Budapest University of Technology and Economics, Faculty of Chemical Technology and Biotechnology, Department of Physical Chemistry and Materials Science, Surface Chemistry Group, Műegyetem rkp. 3., Budapest, 1111 Hungary; klaszlo@mail.bme.hu (K.L.); shereen.farah@mail.bme.hu (S.F.)

[3] Budapest University of Technology and Economics, Faculty of Chemical Technology and Biotechnology, Department of Physical Chemistry and Materials Science, Soft Matters Group, Műegyetem rkp. 3., Budapest, 1111 Hungary; bgyarmati@mail.bme.hu

* Correspondence: mmolnar@mail.bme.hu; Tel.: +36(1)/463-2347

Table S1. Electric conductivity and pH values measured in the assembled test systems after 24 and 48 h of exposure reported from n = 3 determinations per sample. Significant differences when compared to the control are marked by bold italics at each time point

	**Graphene oxide concentration [mg/L]**								
		**Control**			**3.125**			**6.25**	
	**0 h**	**24 h**	**48 h**	**0 h**	**24 h**	**48 h**	**0 h**	**24 h**	**48 h**
pH	7.99±0.03	8.05±0.03	8.09±0.02	8.17±0.03	8.20±0.02	8.21±0.04	8.14±0.01	8.16±0.03	8.18±0.01
EC [µS/cm]	583±8	586±11	588±8	***476±15***	***477±6***	***482±16***	***480±6***	***482±8***	***490±7***
		**12.5**			**25**			**50**	
	0 h	24 h	48 h	0 h	24 h	48 h	0 h	24 h	48 h
pH	8.00±0.01	8.02±0.03	8.01±0.0	7.80±0.03	7.81±0.05	7.89±0.07	7.75±0.01	7.80±0.02	7.88±0.04
EC [µS/cm]	***490±13***	***493±8***	***492±11***	***486±7***	***490±10***	***491±6***	***485±6***	***485±5***	***487±11***

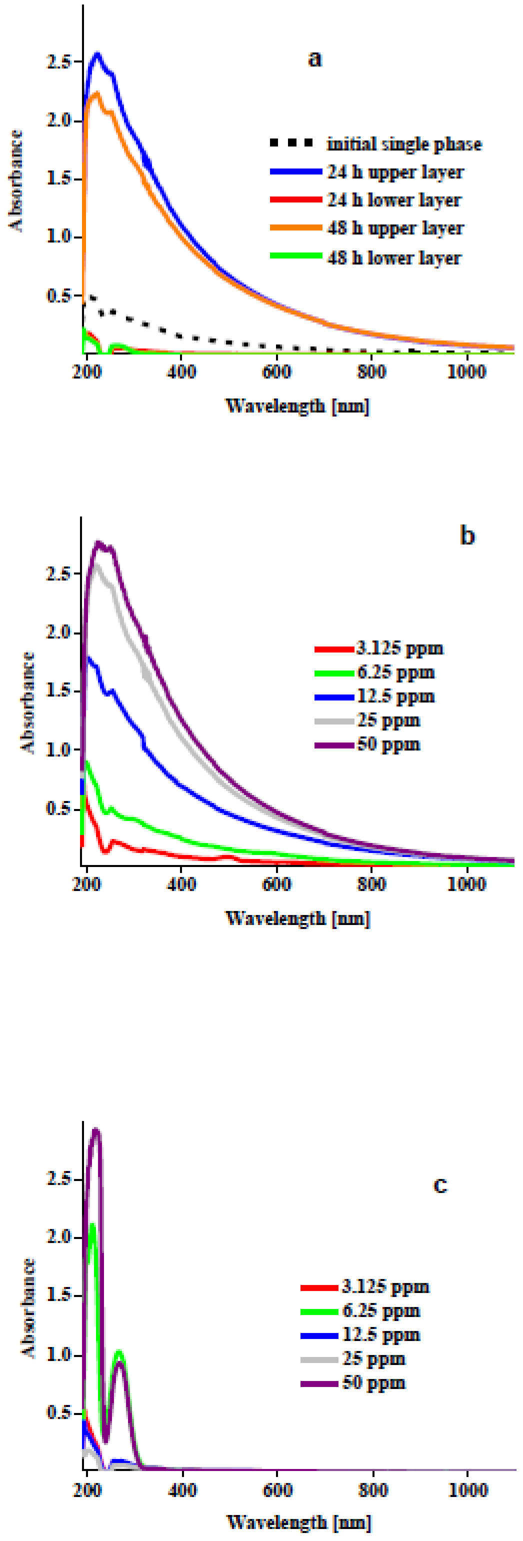

Figure S1. The fate of the GO particles in the test medium followed by UV-Vis spectrometry (a) in the sample with 25 ppm initial GO concentration; (b) in the lower (GO rich) layers of the samples with different initial GO concentration; (c) in the GO depleted (upper) layers of the samples with different initial GO concentration.

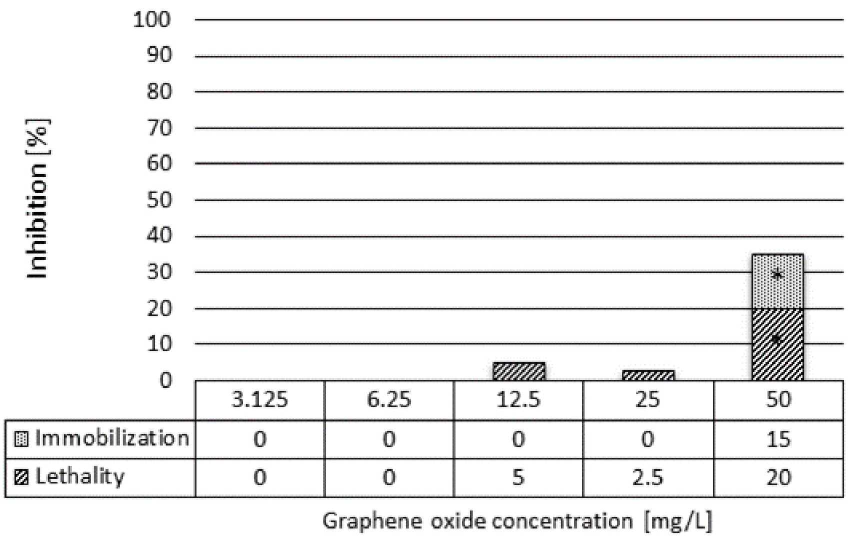

Figure S2. The effect of graphene oxide on the survival and swimming ability of *D. magna* after 48 h of exposure. Standard deviation values were less than 10% in all cases. Significant effect compared to control is marked by asterisk (*).
